# Generalized Anxiety Disorder and Hypoglycemia Symptoms Improved with Diet Modification

**DOI:** 10.1155/2016/7165425

**Published:** 2016-07-14

**Authors:** Monique Aucoin, Sukriti Bhardwaj

**Affiliations:** Canadian College of Naturopathic Medicine, 1255 Sheppard Avenue E., Toronto, ON, Canada M2K 1E2

## Abstract

Observational evidence suggests that a relationship may exist between high glycemic index diets and the development of anxiety and depression symptoms; however, as no interventional studies assessing this relationship in a psychiatric population have been completed, the possibility of a causal link is unclear. AB is a 15-year-old female who presented with concerns of generalized anxiety disorder and hypoglycemia symptoms. Her diet consisted primarily of refined carbohydrates. The addition of protein, fat, and fiber to her diet resulted in a substantial decrease in anxiety symptoms as well as a decrease in the frequency and severity of hypoglycemia symptoms. A brief return to her previous diet caused a return of her anxiety symptoms, followed by improvement when she restarted the prescribed diet. This case strengthens the hypothesis that dietary glycemic index may play a role in the pathogenesis or progression of mental illnesses such as generalized anxiety disorder and subsequently that dietary modification as a therapeutic intervention in the treatment of mental illness warrants further study.

## 1. Introduction

Generalized anxiety disorder (GAD) is a common and often disabling disease. It is characterized by fear, tension, and excessive worries regarding common events or problems for a minimum of six months [1] and is accompanied by physical symptoms such as heart palpitations, muscle tension, and chest tightness. Patients may experience nonspecific symptoms such as irritability and difficulty concentrating in addition to deterioration in the quality of their social, work, and personal experiences [1, 2]. Traumatic life experiences, dysfunctional serotonin, and epinephrine neurotransmitter systems and pernicious genetic influences are potentially involved in the development of GAD [3]. It is estimated that the lifetime prevalence is between 4.3% and 5.9% [1, 3] and 40–67% of patients experience comorbid depression [3].

Conventional treatment of anxiety involves cognitive behavioural therapy in combination with pharmacologic interventions such as serotonin-norepinephrine reuptake inhibitors (SNRI), selective serotonin reuptake inhibitors (SSRI), or benzodiazepines [2, 3]. Despite appropriate treatment, many GAD patients cannot successfully achieve short term or long term remission. After five years, remission rates may remain as low as 38% [2]. Thus, the need for nonpharmacological approaches is evident [4].

Several studies suggest a relationship between improved nutrition and better mental health outcomes, both in general and adolescent populations. Epidemiological studies, including a recent meta-analysis, have shown that a diet including fruits, vegetables, fish, olive oil, nuts, and legumes may reduce the chance of developing depression [4, 5]. A prospective study found that a Mediterranean dietary pattern at baseline reduced the risk of depressive episodes 16.5 years later [6].

Despite evidence correlating a healthy diet with the maintenance of mental health, a trend towards a reduction in the quality of adolescent diets has been observed at the global level. This trend is characterized by an increased intake of fast foods, fried foods, sweets, refined grains, processed meat, and a reduction of fruit/vegetable intake [7]. The consumption of this Western-style diet has been independently associated with a greater risk for the development of anxiety and depression [7] and associated with a decline in the mental health status of adolescents [6]. Another study found an increased likelihood of psychological problems in adolescents associated with diets high in unhealthy foods [7].

A number of possible mechanisms have been proposed for the effect of diet on mental health including a role of inflammation and oxidation [6], deficiency of micronutrients such as folate, zinc, and magnesium [7], and glycemic balance.

The increase in global consumption of refined foods such as sweetened beverages, pastries, and refined carbohydrates impacts dietary glycemic index (GI), a measurement of the rate of blood glucose generation from specific food items. Glycemic load (GL) reflects both the glycemic index value and the total carbohydrate content of the food. Foods with a higher GI or GL create greater increases in blood sugar [8].

A recent cohort study showed that increasing odds of depression and anxiety have been associated with the consumption of foods that have a progressively higher GI and this relationship is maintained after controlling for micronutrients known to play a role in mental health [8]. Cross-sectional studies have demonstrated a correlation between the occurrence of depression/stress and higher intake of sweet foods [9] and a large prospective cohort study showed a positive correlation between a diet high in sweet desserts and refined grains and the risk for depression. Conversely, it was found that high consumption of fruits and vegetables, which contain fiber that lowers GI, was associated with a lower risk for depression [9].

In addition to the observational studies, a limited number of interventional studies have explored a potential relationship between blood sugar balance and emotional and cognitive health. One clinical trial assigned healthy overweight subjects to high or low GI diets and found that the high GI diet resulted in worsening mood scores [10]. Two studies assessed the impact of high and low GI meals on cognitive performance in adults with type 2 diabetes and children, respectively, and found that the higher GI meal was related to poorer cognitive function [11, 12]. Conversely, two other intervention studies comparing high and low GI diets in patients with diabetes did not find a change in subclinical depression or cognitive function [9]. Despite this evidence that a relationship between dietary GI and emotional and cognitive functioning may exist, no interventional studies have assessed the impact of a low GI or low GL dietary intervention in psychiatric patient populations in order to assess causality of the relationship.

## 2. Case Presentation

AB is a 15-year-old female student of south-Asian descent. She presented with concerns of anxiety and symptoms of hypoglycemia as well as difficulty concentrating, fatigue, headaches, asthma, and frequent urinary and vaginal infections.

Her anxiety met criteria for GAD and she rated the intensity of anxiety as 8/10, with 10 being the highest level of anxiety possible. The anxiety started three months prior to the initial appointment and had worsened in the previous month. She described excessive worry that was difficult to control and impacted her daily functioning by causing her to be absent from school on several occasions. She experienced a number of somatic symptoms including heart palpitations, shakiness, discomfort in her stomach, and muscle tension. In response to the anxiety symptoms, she would eat foods like chocolate, chips, or fruit. AB was working with a counsellor to manage the anxiety symptoms and was finding some benefit.

AB had experienced episodes suggestive of hypoglycemia since 12 years of age. The symptoms included muscle weakness and shaking, headaches, nausea, anxiety, and loss of concentration. Her symptoms were ameliorated by eating sweet foods. AB reported that her hypoglycemia was at its worst at 12 years of age when she had to eat a granola bar hourly in order to concentrate.

### 2.1. Clinical Findings

A diet history revealed the following typical daily food intake:

- Breakfast: fruit smoothie containing fruit, fruit juice, and water.
- Morning snack: bagel with margarine.
- Lunch: pasta or white rice with vegetables.
- Afternoon snack: granola bar or cookies or gummy candies.
- After school meal: white pasta; it may include meat.
- Dinner: white rice or spaghetti; it may include meat.
- Evening snack: cookies and toast.
- Beverages: 2 liters of water, 1 cup of juice, 1 cup of lactose-free milk, and 1 cup of tea.

### 2.2. Past Medical History

Due to her difficulty concentrating, AB was prescribed dextroamphetamine 5 mg. While she found that it improved concentration, this medication caused her to lose weight. As the patient's weight was initially on the lower end of normal (weight: 115 lb., height: 5′6′′, and BMI: 18.6) the dose was reduced to 2 to 3 times per week, as needed.

### 2.3. Psychosocial History

AB lives with her mother, father, and sister. AB's sister experienced mental health concerns in the previous years which created elevated stress levels in the family home.

### 2.4. Diagnostic Focus and Assessment

Repeated assessments of random and fasting blood glucose and screening physical examination were within normal range.

### 2.5. Therapeutic Approach

At the initial visit, the following dietary plan was prescribed:

- Breakfast: it includes a smoothie containing fruit, water, 1 scoop of protein powder, and 1 tablespoon of flax seeds or olive oil.
- Lunch and dinner: they include a serving of protein (meat, legume, and soy) and a serving of vegetables.
- Snacks: they include protein when possible (e.g., apple with sunflower seed butter, vegetable sticks with hummus, and pumpkin seeds).
- Continue to eat carbohydrate-containing snacks as needed for the management of hypoglycemia symptoms.

She was asked to follow these dietary guidelines daily until the follow-up visit. While eggs, nuts, and fish would have normally been recommended as well, the patient had an anaphylactic allergy to these foods.

### 2.6. Follow-Up and Outcomes

At the first follow-up, four weeks later, AB reported that she had complied with the dietary plan since the previous visit. She reported a significant decrease in anxiety (4 to 5/10), as well as improved energy, less-frequent and less intense hypoglycemia symptoms, and fewer headaches (once per week compared to daily) in addition to improved concentration and mood. She required fewer snacks during the day and decreased her intake of granola bars, cookies, and candies (1-2 servings per day). She also reported a cessation of chronic vaginal discharge. The substantial improvement in her anxiety symptoms prompted AB to temporarily discontinue her counselling sessions.

At a subsequent follow-up visit four weeks later, AB reported that she had briefly reverted back to her original diet for a period of one week and experienced a worsening of anxiety symptoms within one day. After returning to the dietary intervention prescribed, her anxiety symptoms decreased within two days.

## 3. Discussion

This case illustrates an example of improved anxiety and hypoglycemia symptoms in response to changes in the macronutrient composition of the diet. It suggests that dietary GI and blood sugar balance, within a physiologic range, may play a role in the development or clinical progression of anxiety. Subsequently, modifying the diet by reducing the consumption of refined carbohydrates and including more protein, fat, and fiber may be beneficial.

The proposed relationship is supported by observational studies demonstrating a relationship between higher GI/GL and increased incidence of mental illness as well as a potential mechanism. High GI foods result in an increase in blood glucose levels; however, a large compensatory insulin release may result in reactive hypoglycemia. Hypoglycemia is associated with an acute increase in epinephrine [13] which contributes to neuropsychiatric symptoms including anxiety [14] and symptoms associated with anxiety such as shakiness, sweating, and heart palpitations. Induction of hypoglycemia in a laboratory setting has a negative impact on mood, hedonic tone, and energy levels as well as an increase in tense arousal [15]. The presence of both anxiety and hypoglycemia symptoms in AB's case, along with their concurrent response to treatment, lends support to the hypothesis that these conditions may be related.

However, it is unclear if other macronutrient or micronutrient changes were, at least in part, responsible for the patient's symptom improvement as she had made several changes concurrently. A recent study found that the individuals in the highest third of GI had lower intake of magnesium, vitamin B6, vitamin B12, omega-3 fatty acids, and protein. Evidence shows that all of these nutrients may play a role in the prevention of mental illness [9].

Evidence suggests that adequate intake of protein may be required in the maintenance of mental health. Dietary protein provides amino acids, eight of which are essential. They cannot be synthesized by the body and must be supplied through the diet. Amino acids form the building blocks of neurotransmitters. For example, the synthesis of serotonin requires the essential amino acid tryptophan and dopamine synthesis requires tyrosine, which is produced from the essential amino acid phenylalanine. The lack of tyrosine and tryptophan leads to deficiencies of the respective neurotransmitters and has been associated with psychiatric disturbances [16].

Micronutrient factors are also involved in neurotransmitter synthesis and may be involved in the development of mood and anxiety disorders. As in this case, a diet low in meat may be low in vitamin B12 and a diet low in green vegetables may lack folate. Because both nutrients are important for neurotransmitter synthesis [17], dietary intake may play a role in the development or progression of mood and anxiety disorders.

Dietary fat may also play a role in mental health. The dry weight of the brain is 60 percent fat [18] and low levels of omega-3 fats and cholesterol are significant risk factors for major depression and suicide [19]. Omega-3 fats, which cannot be produced endogenously and must be obtained from the diet, affect the serotonergic system [19] and an inverse relationship between dietary omega-3 fatty acids and anxiety disorders has been demonstrated [7]. Preliminary evidence suggests that omega-3 supplementation may be beneficial in the treatment of anxiety [20].

Despite these other potential factors, the concomitant improvement in both anxiety and hypoglycemia symptoms suggests that blood sugar balance may have been contributing to AB's anxiety disorder and that improvement in blood sugar management, as seen in the reduction of hypoglycaemic symptoms, may have been a significant factor in the response to treatment. In conjunction with the observational data [13], this may strengthen our understanding of the relationship between suboptimal blood sugar regulation, GI/GL, and mental illness.

One strength of this case report is that the dietary modifications were the only intervention initiated at the time during which the improvement in symptoms was observed. As well, the brief return to the patient's previous diet and subsequent worsening of symptoms lends support to the likelihood of a causal relationship.

Some limitations exist as well. No validated assessment tool was used to monitor change in anxiety symptoms, only the patient's subjective report. Additionally, the patient was receiving counselling concurrently.

Blood sugar balance and dietary GI/GL appear to be important factors in a number of chronic illnesses. A recent meta-analysis found significant positive associations between higher GI or GL and higher incidence of diabetes, coronary heart disease, gallbladder disease, breast cancer, and all diseases combined [21]. As such, implementing a diet lower in refined carbohydrates in the treatment of anxiety may have substantial additional benefit with respect to risk of chronic disease.

## 4. Conclusion

Dietary carbohydrates may be significantly related to emotional and cognitive symptoms such as anxiety and difficulty concentrating. Further research into the potential role of GI in the pathogenesis and progression of mental health concerns in addition to the utility of dietary interventions in the treatment of these conditions is warranted.

## References

[1] Martin J. L. R., Sainz-Pardo M., Furukawa T. A., Martin-Sanchez E., Seoane T., Galan C. (2007). Review: benzodiazepines in generalized anxiety disorder: heterogeneity of outcomes based on a systematic review and meta-analysis of clinical trials. *Journal of Psychopharmacology*.

[2] Lalonde C. D., Van Lieshout R. J. (2011). Treating generalized anxiety disorder with second generation antipsychotics. *Journal of Clinical Psychopharmacology*.

[3] Bandelow B., Boerner R. J., Kasper S., Linden M., Wittchen H.-U., Möller H.-J. (2013). The diagnosis and treatment of generalized anxiety disorder. *Deutsches Arzteblatt International*.

[4] Opie R. S., O'Neil A., Itsiopoulos C., Jacka F. N. (2014). The impact of whole-of-diet interventions on depression and anxiety: a systematic review of randomised controlled trials. *Public Health Nutrition*.

[5] Lai J. S., Hiles S., Bisquera A., Hure A. J., McEvoy M., Attia J. (2014). A systematic review and meta-analysis of dietary patterns and depression in community-dwelling adults. *American Journal of Clinical Nutrition*.

[6] Ruusunen A., Lehto S. M., Mursu J. (2014). Dietary patterns are associated with the prevalence of elevated depressive symptoms and the risk of getting a hospital discharge diagnosis of depression in middle-aged or older Finnish men. *Journal of Affective Disorders*.

[7] Kulkarni A. A., Swinburn B. A., Utter J. (2015). Associations between diet quality and mental health in socially disadvantaged New Zealand adolescents. *European Journal of Clinical Nutrition*.

[8] Gangswisch J. E., Hale L., Garcia L. (2015). High glycemic index diet as a risk factor for depression: analyses from the Women's Health Initiative. *The American Journal of Clinical Nutrition*.

[9] Haghighatdoost F., Azadbakht L., Keshteli A. H. (2016). Glycemic index, glycemic load, and common psychological disorders. *The American Journal of Clinical Nutrition*.

[10] Cheatham R. A., Roberts S. B., Das S. K. (2009). Long-term effects of provided low and high glycemic load low energy diets on mood and cognition. *Physiology and Behavior*.

[11] Ingwersen J., Defeyter M. A., Kennedy D. O., Wesnes K. A., Scholey A. B. (2007). A low glycaemic index breakfast cereal preferentially prevents children's cognitive performance from declining throughout the morning. *Appetite*.

[12] Papanikolaou Y., Palmer H., Binns M. A., Jenkins D. J. A., Greenwood C. E. (2006). Better cognitive performance following a low-glycaemic-index compared with a high-glycaemic-index carbohydrate meal in adults with type 2 diabetes. *Diabetologia*.

[13] Sejling A.-S., Kjær T. W., Pedersen-Bjergaard U. (2015). Hypoglycemia-associated changes in the electroencephalogram in patients with type 1 diabetes and normal hypoglycemia awareness or unawareness. *Diabetes*.

[14] Paine N. J., Watkins L. L., Blumenthal J. A., Kuhn C. M., Sherwood A. (2015). Association of depressive and anxiety symptoms with 24-hour urinary catecholamines in individuals with untreated high blood pressure. *Psychosomatic Medicine*.

[15] Gold A. E., MacLeod K. M., Frier B. M., Deary I. J. (1995). Changes in mood during acute hypoglycemia in healthy participants. *Journal of Personality and Social Psychology*.

[16] Sathyanarayana Rao T., Asha M. R., Ramesh B. N., Jagannatha Rao K. S. (2008). Understanding nutrition, depression and mental illnesses. *Indian Journal of Psychiatry*.

[17] Papakostas G. I., Petersen T., Mischoulon D. (2004). Serum folate, vitamin B12, and homocysteine in major depressive disorder, part 2: Predictors of relapse during the continuation phase of pharmacotherapy. *Journal of Clinical Psychiatry*.

[18] Chang C.-Y., Ke D.-S., Chen J.-Y. (2009). Essential fatty acids and human brain. *Acta Neurologica Taiwanica*.

[19] Huan M., Hamazaki K., Sun Y. (2004). Suicide attempt and n-3 fatty acid levels in red blood cells: a case control study in China. *Biological Psychiatry*.

[20] Su K.-P., Matsuoka Y., Pae C.-U. (2015). Omega-3 polyunsaturated fatty acids in prevention of mood and anxiety disorders. *Clinical Psychopharmacology and Neuroscience*.

[21] Barclay A. W., Petocz P., McMillan-Price J. (2008). Glycemic index, glycemic load, and chronic disease risk—a meta-analysis of observational studies. *The American Journal of Clinical Nutrition*.

